# Conductive Regenerated Cellulose Fibers for Multi-Functional Composites: Mechanical and Structural Investigation

**DOI:** 10.3390/ma14071746

**Published:** 2021-04-01

**Authors:** Zainab Al-Maqdasi, Roberts Joffe, Ayoub Ouarga, Nazanin Emami, Shailesh Singh Chouhan, Anton Landström, Abdelghani Hajlane

**Affiliations:** 1 Department of Engineering Sciences and Mathematics, Luleå University of Technology, 97187 Luleå, Sweden; roberts.joffe@ltu.se (R.J.); nazanin.emami@ltu.se (N.E.); anton.landstrom@ltu.se (A.L.); 2 High Throughput Multidisciplinary Research Laboratory, Mohammed VI Polytechnic University (UM6P), Lot 660—Hay Moulay Rachid, 43150 Benguerir, Morocco; ayoub.ouarga@um6p.ma; 3 Department of Computer Science, Electrical and Space Engineering, Luleå University of Technology, 97187 Luleå, Sweden; Shailesh.chouhan@ltu.se; 4 Laboratory of Crystallography and Materials Sciences, National Graduate School of Engineering of Caen, 6 Boulevard Maréchal Juin, 14000 Caen, France; ahajlane@gmail.com

**Keywords:** regenerated cellulose fibers (RCFs), electroless copper plating, conductive cellulose fibers, mechanical properties, molecular structure, functional composites

## Abstract

Regenerated cellulose fibers coated with copper via electroless plating process are investigated for their mechanical properties, molecular structure changes, and suitability for use in sensing applications. Mechanical properties are evaluated in terms of tensile stiffness and strength of fiber tows before, during and after the plating process. The effect of the treatment on the molecular structure of fibers is investigated by measuring their thermal stability with differential scanning calorimetry and obtaining Raman spectra of fibers at different stages of the treatment. Results show that the last stage in the electroless process (the plating step) is the most detrimental, causing changes in fibers’ properties. Fibers seem to lose their structural integrity and develop surface defects that result in a substantial loss in their mechanical strength. However, repeating the process more than once or elongating the residence time in the plating bath does not show a further negative effect on the strength but contributes to the increase in the copper coating thickness, and, subsequently, the final stiffness of the tows. Monitoring the changes in resistance values with applied strain on a model composite made of these conductive tows show an excellent correlation between the increase in strain and increase in electrical resistance. These results indicate that these fibers show potential when combined with conventional composites of glass or carbon fibers as structure monitoring devices without largely affecting their mechanical performance.

## 1. Introduction

The recent attempts to develop sustainable, biobased materials for advanced industrial applications have targeted both processes and development of materials [1]. From the processes point of view, this might include, among others, reducing hazardous substances or using techniques where energy consumption during the production is lowered. On the other hand, material development involves, apart from the use of biobased resources, improving the materials’ characteristics to compete with conventional materials from fossil fuel. For fiber-reinforced polymer composites, low price and density and reduced tool abrasion of natural fibers are examples of interesting advantages over the traditional glass fibers, when designing fiber reinforced polymer composites. Introducing additional characteristics such as electrical functionality to such materials would set their competitiveness to a much higher level.

The use of manmade regenerated cellulose fibers (RCFs) as an alternative to other natural cellulosic fibers has limited the latter’s inherited disadvantages, such as variations in properties and dependency of the structure on growing and cultivation conditions [2]. However, the use of these fibers in continuous form is still limited to textiles and low-end applications. Compared to flax fibers, Cordenka is a technical grade, compostable [3], viscose-type of cellulose II fibers that are manmade developed from the cellulose of wood pulp and mainly used in applications like tire cords. Studies to determine the usability of these fibers in polymer composite applications have shown promising potential in terms of mechanical properties [4,5,6]. Their stiffness lies in the range of 20 GPa, and they possess a strength of about 800 MPa while demonstrating remarkable elongation at break of more than 10% [2]. The large elongation at break makes these fibers candidates for improving the impact strength of thermoplastics [7] and thermoset [6] polymers, while maintaining lightweight. Irrespective of their production technique, all RCFs possess crystallite structure (cellulose II structure) oriented along the fiber axes. However, the degree of crystallization and the degree of polymerization depend on the cellulose source and on the processing technique that results in variations of the properties of RCFs with reference to the production process [8]. A higher degree of crystallinity and more crystallographic orientation result in fibers with higher tensile modulus. Surface defects and internal voids contribute to the variation of tensile strength and fiber’s ability to elongate before failure.

Electroless plating process has been used to successfully obtain electrically conductive fibers and fabrics for use in different applications such as in pressure sensors [9] for wearable health devises, in production of cellulose magnetic fibers [10] and in microwave applications [11]. The electroless process is a multistage, chemical reactions that result in deposition of metal particles from ion-containing aqueous solutions on a wide range of substrates. Regardless of the initial conductivity state of the substrate, plating renders them electrically conductive. The process parameters determine the quality of the coating and the final level of conductivity. One of this process’s advantages is producing homogeneous conductive coatings on complex geometries and flexible substrates without applying an electrical potential difference [12].

For some composite applications, such as in construction, it is important not to exceed a specific deflection under instant or prolonged loading times. One way to achieve this is to continuously monitor the structure in situ. Structural health monitoring allows continuous observation of the structure’s state or state of damage to prevent catastrophic failure and to avoid unnecessary regular service interruptions for inspection. One of the currently popular methods used for structural monitoring in fiber-reinforced composites is fiber optics [13]. Such configuration of a monitoring system gives the flexibility to measure different strain distribution based on the placement of the fibers [14]. However, apart from the price considerations, efficiency of the health monitoring system depends to a great deal on its properties and its compatibility with the host structure. Examples of compatibility can be explained by, but is not limited to, the efficiency of load transfer through strong bonding, the resilience compared to the resilience of the monitored structure, and the suitability to the service conditions. Antunes et al. [15] studied the mechanical properties of standard commercial communication optical fibers to investigate their mechanical limits. The study showed that depending on whether or not the protective layer is present, these fibers possess a stiffness and strength range between 16 and 69 GPa and 100 and 200 MPa, respectively, with a maximum elongation at the break between 0.4 and 4%. The lower bounds of this range might limit their suitability to be used for high-performance fiber-reinforced composites where the failure limits exceed these values. However, a similar approach can be followed to develop greener systems that can offer more varied selection options.

Developing more environmentally friendly and electrically conductive fibers from commercially available materials was reported by the authors previously [16]. The fibers showed a significant decrease in electrical resistance after copper plating for 90 min accompanied by a decrease in mechanical properties. The current study aims to investigate the sources of damage for the electroless plated cellulose fibers and to identify where in the process this damage occurs in a more comprehensive way. Differently from the previous plating strategy, the fibers are coated in consecutive steps of surface activation and plating to obtain improved results. The potential of these fibers to be used for sensing application is also investigated and discussed. A tensile test is performed on fibers obtained from the different stages of the process, and an attempt is made to correlate these properties to the structural changes. Moreover, simple micromechanics calculations are performed to estimate the effect of adding the produced conductive tows on the mechanical properties of conventional composites, in case they are used as strain sensors in such structures.

## 2. Materials and Methods

### 2.1. Materials

Materials used for this study are Cordenka 700 Super 3 (from Cordenka, Obernburg, Germany) RCFs (made by a modified viscose process) in the form of yarns. The yarns consist of 1350 single filaments having a continuous length and semicircular cross-section with an equivalent diameter of ~12.5 µm. Electroless copper plating system (Perfekto-PEC660) is a commercial package consisting of three components (A, M, and B) provided by JKEM International in Norrköping, Sweden. The Alkaline Catalyst System (ACS) used for the plating process was also provided by JKEM International. Epoxy resin system used to manufacture the model composites is hot cure Araldite LY556 and Aradure hardener (Huntsman International LLC, Salt Lake City, UT, USA) in a ratio of 80:20 wt/wt, which cures at 80 °C for 8 h.

### 2.2. Methods

**Figure 1:**
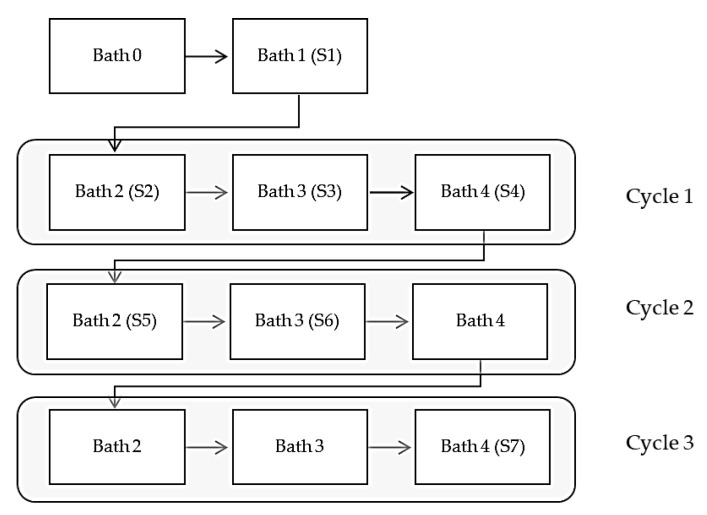
Flowchart depicting stages of the plating process and where along the process, samples were collected.

Plating process (multiple stages): Electroless plating solutions were prepared based on recommendations for the composition and procedure reported in the previous work [16]. To prepare the samples 110 mm long RCF tows/yarns were used. The four baths were prepared, and the tows were immersed in each bath for the specified times shown in Table 1. The initial trials of coating resulted in severe shrinkage of the fibers after immersion in the solutions. Thus, the treatment was performed on tows wound up around a stiff plastic cylinder to prevent shrinkage and allow at the same time for the fibers to be adequately exposed to the solutions to obtain uniform coating. After immersion in the last bath (plating bath, Bath #4) for 30 min, tows were re-immersed in the second, third and fourth bathes two more times resulting in a total of 55–60 min of consecutive plating. The residence time in the plating bath was decided based on a visual assessment of the coating state/quality. Gentle stirring was applied to the baths to ensure homogeneous concentration within the solution. The idea behind the repetitive plating process is that any individual fibers or parts of fiber, which might not have been plated in the first round would be activated and plated in the following cycles. For the determination of the property changes of fibers along the process, samples were collected after specific stages, dried at room temperature and saved in sealed plastic bags to perform further characterization. The steps of the Cu-electroless plating adopted during the process is depicted in the flowchart of Figure 1, where (S) represents the sample set (4 specimens per set) and was used throughout the paper. Samples used for the initial characterization and the production of model composite were prepared following the same procedure but with replenishing of the plating bath to ensure even better quality of the coating and with total residence time in the plating bath of 90 min. Further in the paper, the latter was referred to as coated regenerated cellulose fibers (CRCFs).

Microscopy: Nikon Eclipse microscope MA200 (Nikon Corporation, Tokyo, Japan) equipped with digital viewing camera was used for the microscopic investigation. In order to investigate the cross-section and the coating thickness, dry tows were first embedded in a slab of Epoxy resin and thoroughly polished for better visualization during examination. The thickness of the copper layer was measured over several fibers around the bundle, and the average value was reported.

Mechanical test on dry tows (quasistatic tensile test): Virgin and treated tows were tested using Instron 4411 universal tester (Instron, Norwood, MA, USA) equipped with a 500 N load cell. Dry tows with a gauge length of 40 mm were gripped between wooden tabs at both ends and strained at a rate of 10%/min until failure. Compliance of the machine was determined by testing two additional lengths of virgin fiber tows (60 and 100 mm) as specified in the ASTM standards [17] and this compliance was taken into account when calculating true stiffness of the tows. Since limited length was available for the coated fiber tows, their true stiffness was determined using the same value of machine compliance used for the non-coated tows. Stiffness was determined from the initial region of the stress-strain curves between the stress interval of 30–130 MPa.

Thermal Stability: Virgin and electroless-plated tows were tested for thermal stability utilizing differential scanning calorimetry (DSC) equipment, METTLER TOLEDO DSC 821 e (METTLER TOLEDO, Columbus, OH, USA). Samples of 5 mg weight were enclosed in a standard aluminum pan (METTLER TOLEDO, Columbus, OH, USA) with a pierced lid and exposed to temperature program in a nitrogen atmosphere. Samples were heated at a rate of 10 °C/min to 200 °C and cooled with the same rate to room temperature, then heated again to 450 °C to examine degradation. For the copper-plated samples (CRCF), fibers from the inner part of the bundle were separated and used for the test. However, it was not guaranteed that the samples were totally copper-free, but this would not affect the results since thermal events of metals start at a higher temperature than temperatures of interest for polymers.

Raman Spectroscopy: Samples were investigated with Raman spectroscopy to study the internal structural changes at the different stages of the electroless process. Raman data were collected with a WITec CRM-200 Raman/fluorescence imaging microscope (WITec, Ulm, Germany) using a He:Ne 633 nm excitation laser. Acquisition time was set to 10 s, and an average of 10 spectra is presented. As mentioned earlier, samples collected from the inner part of the bundle were examined.

**Figure 2:**
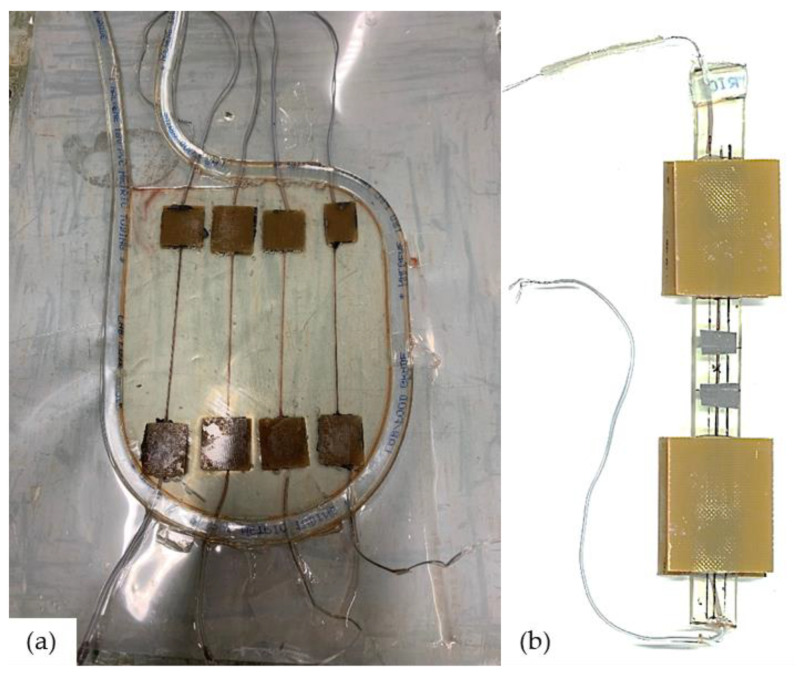
(**a**) Overview of the coated regenerated cellulose fiber (CRCF) tows embedded in epoxy resin at the manufacturing and an (**b**) example of the tabbed specimen of the model composite.

Manufacturing of the model composite: The model composite was made by embedding single conductive tow in Epoxy resin. Due to the importance of the connection to the electrical measurements, it is necessary to produce a firm connection between the coated fibers and the measuring device. Preliminary trials of direct twisting of tows with electric wires resulted in weak points that have caused a premature failure at the connection during the test. Alternatively, conductive carbon cement (PLANO GmbH, Wetzlar, Germany) was used to glue the fibers to the wires between two tabs of generic glass fiber weave/Epoxy laminates (Nordbergs Tekniska AB, Vallentuna, Sweden) available for this purpose that were further secured with epoxy resin during the impregnation. Tabbed, wired tows were aligned in a sealed aluminum mold for resin casting. Measured amounts of the resin (80A:20B wt/wt) were mixed and degassed for 15 min before being poured into the mold and cured at 80 °C for 8 h. After demolding, samples were cut into 13 mm wide, 100 mm long rectangles with the centralized coated tow connected to wires from both ends. Specimens were tabbed again using the same tabbing material and epoxy adhesive leaving 40 mm as a sample gauge length at the center of which is the extensometer’s 12.5 mm gauge length. With such a setup, the connection points ended up outside the gauge length of the sample. An overview of the manufacturing setup and sample configuration is presented in Figure 2.

Strain–electrical resistance test: A single-tow composite was tested for change in electrical resistance under applied mechanical loading. Samples attached to wires were connected to a reference 3000 Potentiostat/Galvanostat (Gamry Instruments Inc, Warminster, PA, USA) where a constant current of 1 mA was supplied while the sample being mechanically loaded. Strain was monitored by means of standard Instron clip-on extensometer 2620-601 (Instron, Norwood, MA, USA). Change in resistance was monitored by measuring the drop in voltage across the sample ends. The tensile load was applied through a universal Instron 3366 tester (Instron, Norwood, MA, USA) equipped with a 10 kN load cell. The loading rate was set to a rather low value (0.5%/min) in order to capture the change in electrical resistance. Synchronized data sampling allowed correlating the data collected from the two setups. Different loading ramps were applied to investigate the electromechanical response of the composite.

## 3. Results and Discussion

Electroless plating of the RCF tows resulted in electrical sheet resistance of tows of around 0.3 ± 0.04 Ω/50 mm sample length.

**Figure 3:**
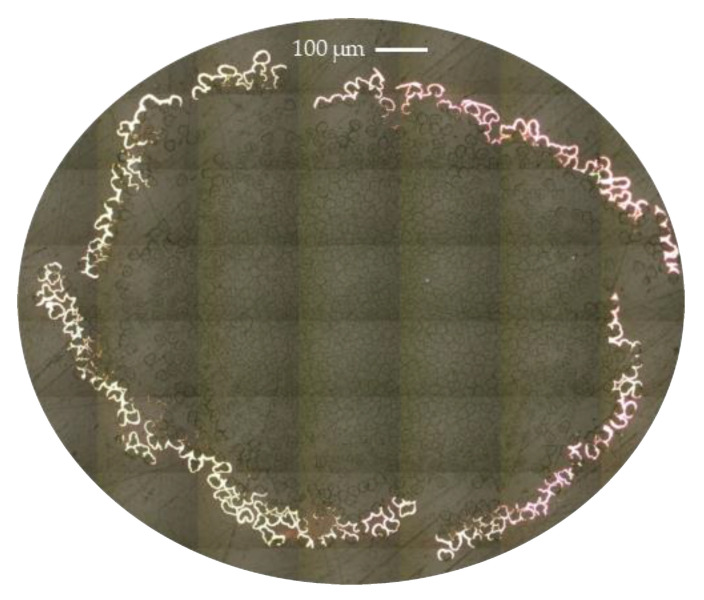
Cross-section of coated tows (CRCF). The copper is visible as bright lines on the perimeter of the tows.

Figure 3 shows the cross-section of a fiber tow after the coating. It can be seen that the copper deposition is limited to the outermost fibers of the tow. When copper starts to deposit, it is easily deposited on the surface of the tow forming a thin continuous layer that blocks the access to the inner fibers of the tow (note that the discontinuity seen in the figure is resulting from the post-processing and handling of the bundle during immersion in the resin and is considered as human error). With progression in exposure time, this layer gets thicker with more deposited particles on top than penetrating inside the bundle. An average thickness of the copper layer at random positions around the bundle was around 6 µm. This value was almost twice as large as reported in our previous work when the deposition was performed in a single continuous step for the same duration of time [16]. Despite the relative slow deposition of copper by the electroless plating (compared to, e.g., the electrolyte process), it is reported to result in a homogeneous deposition on both conductive and non-conductive substrates [12]. Figure 3 also shows that the fibers were not typically circular, but they had more cherry-like shape, which was retained after the coating process.

**Figure 4:**
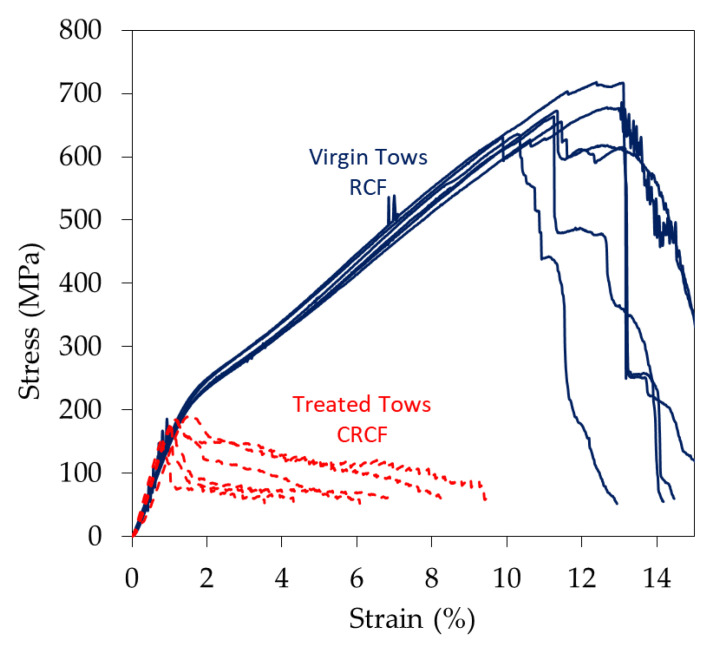
Stress-strain curves of the tows tested in tension before (solid blue line) and after (CRCF) (dotted red line) coating by electroless copper plating.

Figure 4 shows the stress-strain curves of the virgin RCF and tows after the coating process (CRCF). Typical stress-strain curves of the RCF are represented by three main stages: an initial linear region, a yield point and second linear plastic deformation [2]. The very first part where the curve has a tail of non-linearity is attributed to the clamping errors and the slack in fibers and can be compensated following the practice described in ASTM [17]. The initial linear part represents the Hookean behavior of the fibers from which the stiffness of the bundle is determined. Degree of crystallinity and the orientation of crystallite in cellulose fibers are the major contributors to increased mechanical properties in return for the negative effect of void content. Studies on RCF with different orientation of cellulose chains showed the dependency of their stiffness and strength on the degree of orientation with a clear increase of stiffness and strength when the chains are oriented along the axis of the fiber at the same crystallinity content [18]. Then follows a yield point after which a strain hardening semi-linear region occurs. With the aid of X-ray diffraction (XRD) measurements, it was found that the orientation factor of the cellulose molecules increases with the increased straining of the fibers [19]. This response is also the effect of reorientation of the non-crystalline part of the regenerated cellulose [20], which is usually a large portion of the molecular structure especially for viscose fibers (crystallinity is rarely higher than 50%) [21].

The effect of the treatment on the tows can also be seen when examining curves of CRCF in Figure 4. Stiffness had slightly increased by the effect of deposited copper while a considerable decrease in the strength is noticed (drop from 650 to below 200 MPa). The degradation is represented by the loss of the second semi-linear part of the stress-strain curves where the linearity after the yield no longer exists. However, the strain at failure is relatively retained. The interaction with chemicals during the plating process has possibly resulted in the loss of orientation of the cellulosic chains and the relaxation of the stretched chains during the process, leaving the chains more accessible to chemicals and thus weaker. Similar results were noticed for conductive linen fabric subjected to an acidic environment where it has been reported to lose a significant percentage of its strength [22]. Average values of properties of at least six tested specimens are presented in Table 2.

**Figure 5:**
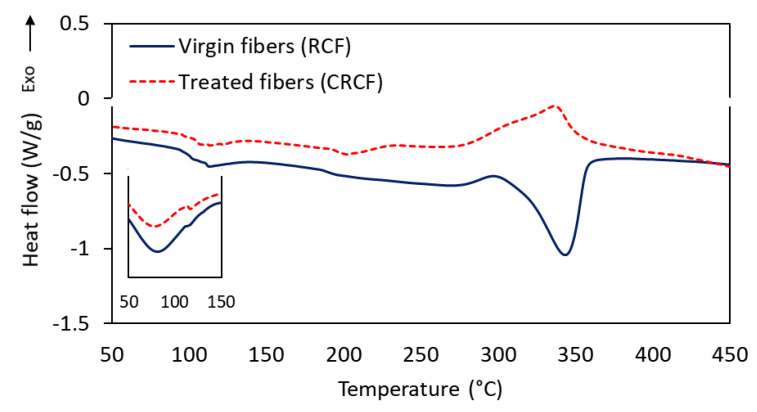
Differential scanning calorimetry (DSC) thermograms of the virgin regenerated cellulose fiber (RCF) (solid line) and electroless copper-plated CRCF (dotted line). The inlet in the figure is for the moisture evaporation peak at the first heating ramp.

DSC was performed on these two types of fibers (RCF and CRCF) to investigate the effect of the treatment on the thermal behavior of the fibers. Figure 5 shows the thermograms with the most interesting thermal events. The first broad endothermic event having a peak at around 100 °C was associated with the loss of adsorbed water on the samples. The inlet in the figure shows the size of the peak at the first heating ramp. At the second heating cycle, the size of the peak was reduced, but not entirely eliminated. Removing the large peak helped improve observation of the subsequent events as they become more pronounced. Following the moisture evaporation, the virgin RCF fibers exhibited a well-defined endothermic event with a peak at 350 °C, which was associated with decomposition and depolymerization reactions of cellulose and the decomposition of the subsequently formed complex products [23,24]. This peak shifted to much lower temperatures for the treated fibers with less energy required for the decomposition. The broad endothermic event had a peak around 204 °C and another less defined around 270 °C. Viscose fibers treated with boric compounds have shown similar degradation behavior and shift in the degradation temperature attributed to the elimination of cellulose intermolecular hydrogen bonding, which accelerates the decrystallization of the fibers [23]. A small very broad exothermic peak around 216 °C in the treated fibers can be attributed to the weak events of recrystallization of traces of copper particles that might have ended up in the sample [25]. The last exothermic peak in the treated fibers represents an overlap of several processes occurring at that range of temperatures [26]. This peak is attributed to the charring process, which shows another difference from the non-treated fibers where the cellulose mainly undergoes quick devolatilization reaction [27].

**Figure 6:**
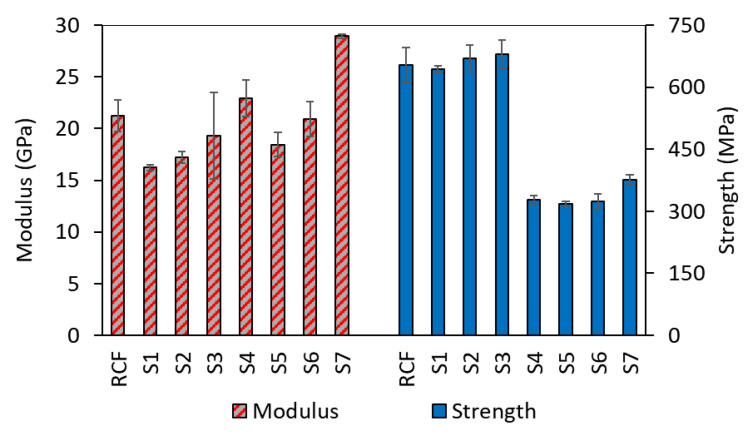
Modulus and strength of the samples at different stages of the plating process. Sample names and their corresponding stages are presented in Figure 1.

It is of interest to inspect at which stage during the electroless process this loss of mechanical properties occurs, and to investigate the leading cause for failure. Samples taken at different stages of the coating process were used for this purpose, and their mechanical properties (average of 4 values) are presented in Figure 6. It can be seen that immediately at the first stage, there is a drop in the stiffness with reference to the virgin fibers (23%). This decrease is attributed to the contact with moisture in the solutions. Moisture has been shown to have a drastic effect on the properties of RCF [28]. There seems to be a slight increase in both the stiffness and the strength within the first three stages of the process, but it was not of statistical significance according to the statistical analysis of variance (ANOVA). At the fourth stage where the copper was deposited, the largest drop in the strength was witnessed while the first significant improvement in the stiffness occurred. The following stages did not seem to have further effect on the strength, but the second deposition step contributed to more of a stiffness increase in the tows. It might be the case that the deposited copper around the bundle at the first coating round is acting like a protective layer preventing the chemicals from reaching the core of the tows and only more copper is deposited on top of the previously deposited layer causing the additional increase in stiffness. These results confirm the previously obtained results on single fibers [16], suggesting increasing the stiffness by increasing the residence time in the plating bath or the number of deposition steps without further compromising the strength. However, replenishing the bath (results of CRCF in Table 2) resulted in tows with lower strength than the method without bath renewal. It is worth noting that after a long period of time from producing the samples, they showed changes in their properties due to handling and due to the disintegration of the copper layer from the surface of the fibers. This made it challenging to perform additional tests that can be compared with those of initially produced samples.

**Figure 7:**
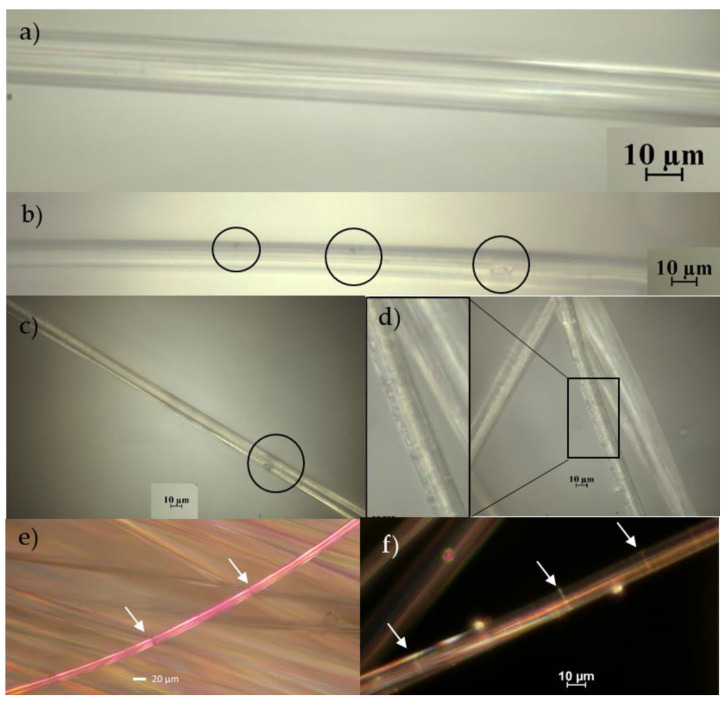
Optical micrographs for virgin (**a**,**b**,**e**) and treated fibers (**c**,**d**,**f**). Images (**e**,**f**) are for fibers within tested tows. The treated fibers are taken from the inner part of the bundle to facilitate examining the bare surfaces of fibers rather than the coating.

Fiber surfaces, and internal molecular structures, were investigated at different stages of the process. In order to avoid examining only the coating layer, fibers from the inner part of the tows were extracted and carefully examined. Micrographs are presented in Figure 7 for virgin RCF and treated fibers. It can be seen that some surface defects in terms of pits (enclosed in circles) are presented on surfaces of both types of samples. For the virgin fibers, these can be inherited manufacturing and handling defects. Similar types of defects were noticed at the surface of the fibers extracted from the other stages as well. However, for the treated fibers (e.g., sample S4 presented here), surfaces show additional types of defects. These defects were in the form of surface roughening, as can be seen from the inlet of Figure 7d, and increased the size of the pits. It can also be seen from the images in Figure 7e,f that stressed fibers developed regions of weak points at multiple locations along the fiber surface (indicated by white arrows). The more stressed the fiber is, the larger the number of these regions becomes. This can cause catastrophic failure for the coated fibers in the case where the coating fails at a region in the vicinity of such a weak point. Considering the increased number of defects on the surface of the treated fibers and the additional risk presented by the coating failure, it is well justified that these fibers fail at such small loads. Since the most damaged fibers are present at the outer part of the tows, the load after initial failure is distributed at continuously decreasing the number of surviving resilient fibers, which explains the relatively large strain at ultimate failure presented in Figure 4. It is worth noting that due to the high stretchability of the virgin fibers, they sustain high loads and fail in a catastrophic way similar to that of a single fiber. On the contrary, coated tows exhibited the usual progressive failure of tows indicating statistical strength distribution (e.g., Weibull distribution), which is governed by the distribution of defects. It should also be mentioned that there was a considerable variation between the surfaces of the different fibers, but the above is a general observation at each stage, which was not statistically evaluated but was correlated to the respective stage.

**Figure 8:**
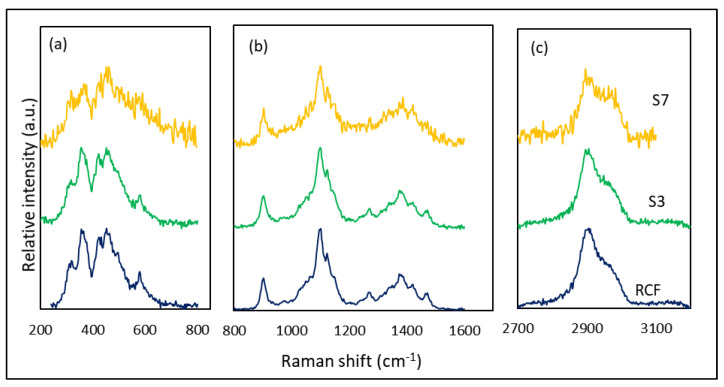
Raman spectra of virgin RCF compared to fibers at different stages of the electroless plating process, at three different spectral intervals. (**a**) 200–800 cm^−1^, (**b**) 800–1600 cm^−1^, and (**c**) 2700–3200 cm^−1^. All peaks are normalized to the maximum peak value at the respective interval; peaks were shifted vertically for presentation purposes.

Raman spectra of the fibers at different stages are presented in Figure 8, together with the spectrum of virgin fibers. In general, virgin fibers show typical spectrum of cellulose represented by the CH, CH_2_ stretch around 2906 cm^−1^, H–C–H and H–O–C bend at 1478 cm^−1^, H–C–C, H–C–O, and H–O–C bend at 1379 and 1334 cm^−1^, C–C and C–O stretch at 1108 cm^−1^, C–O–C in-plane symmetric at 910 cm^−1^, and bends of skeletal C–O–C, C–C–C, O–C–C, and O–C–O at 516–379 cm^−1^ [29]. Since the loss of mechanical properties was associated with the plating stage (as indicated by Figure 6), selected samples from specific stages were analyzed (virgin fibers, S3, S4, and S7) to examine the possible internal structural changes. However, samples taken from stage 4 (sample S4), showed very strong fluorescence that it was not possible to resolve a good quality spectrum. Therefore, sample S7, which undergoes the same steps was selected for the test. It is interesting to notice that spectra of S3 and the virgin RCF did not differ essentially in terms of the present peaks and their positions. No new peaks associated with the chemical modification at that stage were introduced. This can either be due to the selection of the fibers from the inner part of the bundle where the chemical reaction was not very strong; or to the fact that they are overlapping with the reference peaks and are too weak to observe. Another possibility is that these bands are poorly polarizable and are therefore transparent to Raman. However, more distinctive differences in features for the spectrum of sample S7 were observed. In general, the spectrum was broad and less defined than that of virgin RCF. The broadening of the RCF spectra has been associated in the literature to reduced order of the molecular structure [30]. Moreover, relative intensities seem to differ between the spectra of the virgin and treated fibers. Alves et al. [31] have reported four main changes in the relative intensities of the Raman bands related to the structural changes in cellulosic fibers. The decrease in intensity of the peak at 1121 cm^−1^ relative to 1096 cm^−1^ was related to the degree of organization of fibers and was commonly found in amorphous cellulose. Shortening of cellulose chain length due to disruption of glycosidic linkages was observed as a decrease in the ratio of peak intensities at 1096 and 2896 cm^−1^. Moreover, the degree of crystallinity of fibers can be correlated to the peak intensities at 380 and 1096 cm^−1^. Finally, the crystalline arrangement can be assessed by the intensity of highly ordered crystalline cellulose (peak at 1476 cm^−1^) and low organized crystalline cellulose (peak at 1461 cm^−1^). The spectra of S7 show most of these features, more particularly, a decrease in the intensity ratio of the bands at 1476/1461 was noticed (it went from 0.73 in virgin RCF to 0.53 in S7, while no significant change was noticed for S3). This allows the assumption that the crystallinity and the structural order of the fiber have been decreased after the treatment, which might be the reason for the decrease in mechanical properties discussed earlier. The generally less defined features of the spectrum of S7 imply the same conclusion.

## 4. Potential of Coated Fiber for Use as Strain Sensors

**Figure 9:**
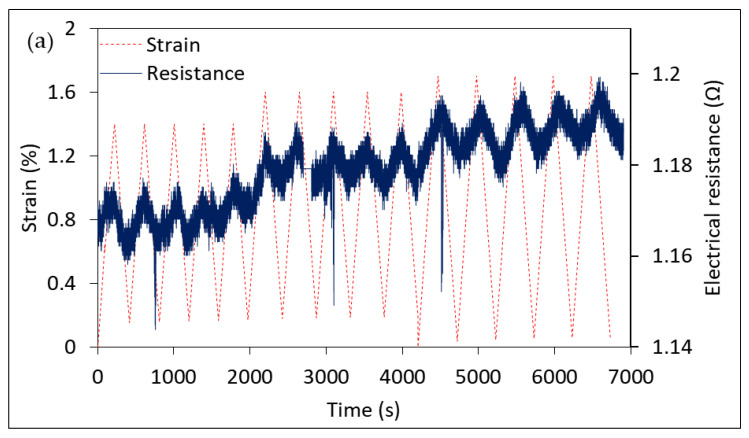

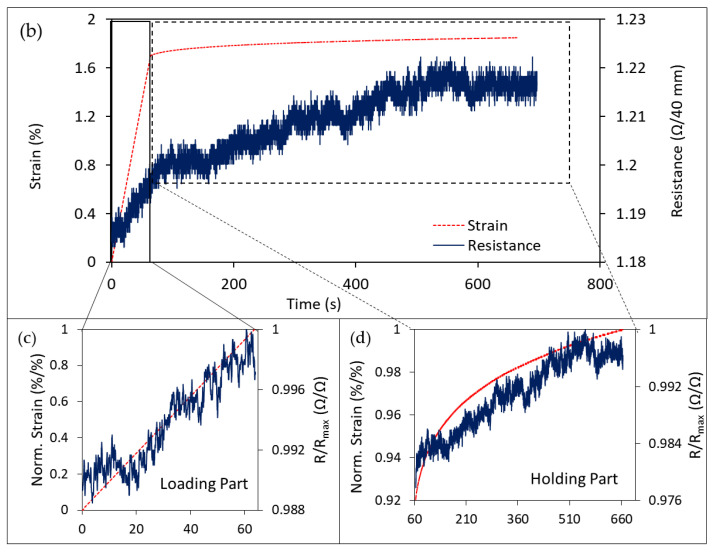
(**a**) Electrical resistance response to the induced mechanical strain in the loading–unloading tensile test at different strain levels and (**b**) electrical resistance response to creep-like test, (**c**,**d**) are the enlarged views of the loading and holding parts in (**b**), respectively, and are normalized with respect to the maximum value at each part.

After characterizing fibers for their mechanical properties and the internal structure, an evaluation of their performance for sensing application was carried out. Typical electroless copper-plated structures used in electroless plated wiring boards have the potential to elongate up to 15% without the loss of conductivity [12]. Manufacturing a successful, durable single tow/epoxy model composite was somewhat challenging. The coating was detaching due to the frequent handling of the tows and the long-term storage. This has reduced the chances to obtain testable samples, and thus one good specimen was used to perform several loading profiles where electrical resistance has been monitored with respect to the applied strain. First, the sample was strained in a cyclic manner between 0% and 1.4% strain for 4 cycles followed by other sets of 4 cycles with strain raised up to 1.6% and 1.7% as shown in Figure 9. As can be clearly seen, these tows’ resistance was increasing as they were being mechanically strained and consistently decrease to initial resistance at load removal. The level of increase was also consistent with the change of the strain level. Further increase of strain from 1.4% to 1.6% or 1.7% results in a subsequent increase in the electrical resistance. Another loading scenario represented by the creep-like load is also shown in the figure. The sample was loaded to 1.7% strain, and the load was held constant for 10 min. Once again, the resistance lines followed the strain behavior nicely. As the load was being held constant, the sample creeps and strain increased nonlinearly with time. The resistance changed at the same rate, taking the shape of the curve in both parts (loading and holding). A correlation coefficient between the strain and resistance data sets was found to be 0.903 and 0.926 for the loading and holding parts, respectively. Note that data in the separate graphs of the two parts were normalized to their initial value at the individual part for clarity. However, these results were obtained from testing a single specimen and need to be validated by manufacturing more samples and checking for the repeatability. Such correlation shows a promising potential for these materials to capture the strain changes and the possibility to use them as sensors within a composite structure.

Using the measured properties and theoretical properties of conventional fibers, simple rule of mixtures, presented in Equation (1), was used to estimate the change in stiffness upon addition of copper-treated regenerated cellulose fibers (CRCF) to conventional composites of carbon fibers (CF) and glass fibers (GF) in the epoxy matrix (EP).

(1) $$E_{C}=E_{f}V_{f}+E_{RCF}V_{RCF}+E_{m}V_{m}$$

**Figure 10:**
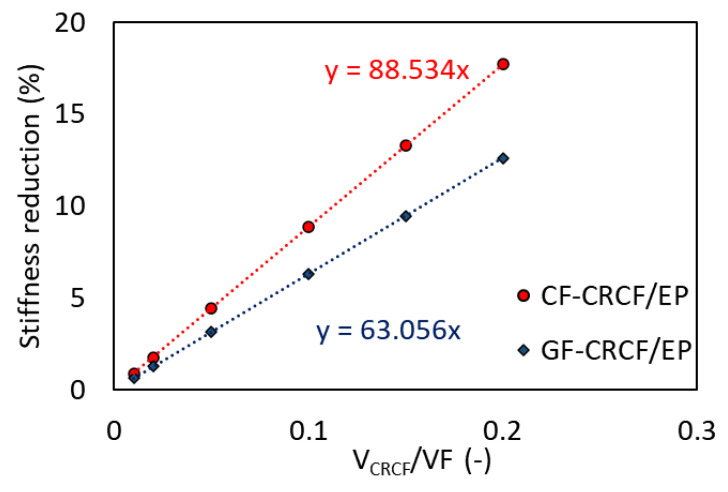
Reduction in stiffness of conventional composites by addition of coated cellulose tows as estimated by the rule of mixtures. The equations on the figure correspond to the trendlines of the data points, where x is the slope of the line.

In Equation (1), E is the stiffness, V refers to the volume fraction, and the subscripts *C*, *f*, *RCF*, and *m* refer to the composite, reinforcing fibers, additional *RCF*, and the matrix, respectively. The volume fraction of the CRCF was varied with respect to a fixed total volume fraction of fibers in the composite (VF = 60%). Theoretical stiffness values used for the estimation are: GF = 70 GPa, CF = 230 GPa, EP = 3 GPa, and the experimentally measured value for CRCF = 24.5 GPa presented in Table 2. Results of the estimation are plotted in Figure 10. As can be seen, the addition of the treated fibers causes a reduction in the total stiffness of the composite since the added fibers have lower stiffness than the removed ones. However, up to a fraction of 20% of added fibers, the maximum stiffness reduction did not exceed 18% for CF/EP and 13% for GF/EP. For sensing application, only small fractions (5%) of such tows were sufficient for the detection purposes, which led to smaller negative effects. Compared to optical fibers, this type of sensors was closer in the dimensions to fibers in the reference composites (especially when properly impregnated and wetted) and thus did not cause large stress concentrations as do larger inclusions. Though, one should bear in mind that these fibers exceeded the strain limits of the conventional composites, but their strength was still not as comparable.

## 5. Conclusions

Electroless copper plating process was used to produce conductive regenerated cellulose fibers. Improved quality coating was achieved by depositing copper in multiple surface activation and plating cycles. The conductive fibers exhibited a decrease in the mechanical properties, which was traced back to the chemicals in the process. Rather than the activation bath, the plating step was the most detrimental stage where the fibers’ strength was reduced by approximately 50%. The stiffness was less severely affected as the initial reduction caused by the wet chemistry did not exceed 23% and was almost wholly retained when the stiff copper layer was deposited. Both surface defects and internal changes in the molecular structure were found to be responsible for the degradation of mechanical performance as was confirmed by optical microscopy, Raman microspectroscopy and differential scanning calorimetry. The treated fibers exhibited larger surface defects (major factor affecting strength) and broader Raman spectra with less defined peaks than that of virgin fibers and exhibited earlier thermal degradation. The internal structure changes were mostly related to the decrease in structure organization and loss of crystalline order. Despite the reduction in the mechanical performance, these fibers showed potential to be used for applications where small amounts of conductive tows were needed, or where conductivity and stiffness were of greater interest than strength. Preliminary results showed a considerable potential to use these fibers for sensing applications. Incorporating a number of coated tows in composites of conventional fibers can act as structure health monitoring devices with a limited adverse effect on the mechanical performance of the host composite that do not exceed 18% based on theoretical estimations.

## Figures and Tables

**Table 1:** Parameters of the plating process. DIW stands for deionized water; RT for room temperature (22 ± 2 °C).

Bath No.	Chemistry	Temperature (°C)	pH	Time (min)	Sample
0	DIW	RT	-	-	-
1	Pre-dip	RT	11–12	1	S1
2	Catalyst ACS 74	40	11.5–12.5	5	S2, S5
3	Reducing bath, H_3_BO_3_	25–30	7	5	S3, S6
4	PEC 660 (A, M, B)	25	-	30, 10, 15	S4, S7

PEC is the commercial name of the electroless chemical package. (H_3_BO_3_) is the chemical structure of Boric acid powder provided by VWR Chemicals in Leuven, Belgium.

**Table 2:** Properties of the tested tows before and after treatment. Values were the average of 5 specimens. Std stands for “standard deviation”, E. Resistance is the “Electrical Resistance”.

Sample	Modulus (GPa)	Std	Strength (MPa)	Std	E. Resistance (Ω/50 mm)	Std
RCF	21.25	1.53	653.93	42.3	-	-
CRCF	24.60	0.09	180.51	10.9	0.3	0.04

## Data Availability

Data sharing not applicable.
